# Gender difference in the effects of interleukin-6 on grip strength – a systematic review and meta-analysis

**DOI:** 10.1186/s12877-018-0798-z

**Published:** 2018-05-08

**Authors:** Alexandra Mikó, László Pótó, Péter Mátrai, Péter Hegyi, Nóra Füredi, András Garami, Anita Illés, Margit Solymár, Áron Vincze, Márta Balaskó, Gabriella Pár, Patrícia Sarlós, Judit Bajor, Judit Tenk, Ildikó Rostás, Erika Pétervári

**Affiliations:** 1 Institute for Translational Medicine, Medical School, University of Pécs, Szigeti út 12, Pécs, 7624 Hungary; 2 Institute of Bioanalysis, Medical School, University of Pécs, Szigeti út 12, Pécs, 7624 Hungary; 3 Department of Translational Medicine, First Department of Medicine, University of Pécs, Szigeti út 12, Pécs, 7624 Hungary; 4 Hungarian Academy of Sciences–University of Szeged, Momentum Gastroenterology Multidisciplinary Research Group, Korányi fasor 8-10, Szeged, 6720 Hungary; 5 Department of Anatomy, Medical School, University of Pécs, Szigeti út 12, Pécs, 7624 Hungary; 6 Division of Gastroenterology, First Department of Internal Medicine, University of Pécs, Ifjúság út 13, Pécs, 7624 Hungary

**Keywords:** Sarcopenia, Inflammation, Interleukin-6, Grip strength, Gender

## Abstract

**Background:**

Aging sarcopenia characterized by low muscle mass with low muscle strength affects men and women differently. The contribution of interleukin-6 (IL-6) to sarcopenia has been suggested based on a negative correlation between plasma IL-6 and muscle function described by some studies. However, no consensus regarding clinically relevant cut-off criteria has been reached. Another question arises whether pooling male and female data is an accurate way to determine the predictive value of IL-6 in sarcopenia. The present meta-analysis was designed to assess: (1) whether plasma IL-6 in aged populations in fact correlates negatively to muscle strength; (2) whether such a correlation exists both in men and in women; and (3) whether plasma IL-6 shows a gender difference in old age.

**Methods:**

We applied the preferred reporting items for systematic review and meta-analysis protocols (PRISMA). We searched PubMed and Embase for papers that reported data on individuals over 65 without inflammatory diseases. We extracted either separate male and female data on plasma IL-6 along with at least one muscle parameter or correlation coefficient between plasma IL-6 and these parameters. Random effect models calculated with DerSimonian and Laird weighting methods were applied to analyze correlation coefficients and gender difference in plasma IL-6. Egger’s test was used to assess the small study effect.

**Results:**

Twenty articles out of 468 records identified were suitable for analyses. Plasma IL-6 correlates negatively with grip strength in mixed populations and also separately in men [− 0.25 with 95% confidence interval (CI): − 0.48, − 0.02] and in women (− 0.14 with 95% CI: − 0.24, − 0.03). However, contrary to expectations, men with better muscle condition have higher plasma IL-6 than women of similar age with worse muscle condition (plasma IL-6 male–female difference: 0.25 pg/mL with 95% CI: 0.15, 0.35).

**Conclusion:**

This is the first study to demonstrate that a higher predictive IL-6 cut-off level should be determined for aging sarcopenia in men than in women.

**Electronic supplementary material:**

The online version of this article (10.1186/s12877-018-0798-z) contains supplementary material, which is available to authorized users.

## Background

Population aging particularly affects women (since they live longer) resulting in the “feminization of old age” [1]. Women are especially affected by “aging sarcopenia”, defined by the European Working Group on Sarcopenia in Older People as a geriatric syndrome characterized by progressive, generalized loss of muscle mass and muscle strength or physical performance without necessary occurrence of any disease [2]. Sarcopenia is of outstanding clinical importance, as it leads to frailty, tiredness, falls, fractures, functional disabilities, comorbidities, higher health care expenditure and premature mortality [3].

Immunosenescence that involves chronic systemic, low-grade inflammation coined as “inflammaging” contributes to the progression of aging sarcopenia [4]. Inflammatory cytokines, e.g. interleukin-6 (IL-6), have catabolic effects on muscle proteins [5]. This mediator is called the cytokine of gerontologists [6]. The main sources of this Janus-faced cytokine with pro- and anti-inflammatory features include immune or endothelial cells and adipocytes [7]. Plasma IL-6 (pIL-6) increases with age both in healthy men and women [8, 9], but gender differences still remain largely unexplored. Body composition, especially visceral fat, also correlates to IL-6. As a myokine, pIL-6 increases for several hours following exercise [10], but chronically active persons have lower pIL-6 [11].

There are controversies regarding IL-6 in the literature. On the one hand, pIL-6 released during muscle contraction inhibits pro-inflammatory cytokines [12]; on the other hand, it is a marker of inflammatory status [4] and a predictor of disability and mortality in the elderly [13]. Sarcopenia could be the link between high pIL-6 and disability. Plasma IL-6 has been shown to be negatively correlated to muscle mass and function (mostly indicated by grip strength) in the elderly [14–28]. Follow-up studies [29, 30] have also suggested a role for pIL-6 as a biomarker of sarcopenia. In contrast, other studies have failed to confirm a similar relationship [9, 20, 24, 25, 31]. Gender differences may be the confounder that explains the controversy. Pooling male and female data may not be an accurate way to determine the predictive value of pIL-6 in sarcopenia. However, gender differences have not been properly analyzed, although this is a clinically relevant question.

A recent meta-analysis has suggested an association of elevated pIL-6 with frailty that has not been confirmed by longitudinal studies [32]. However, one of three longitudinal studies applied too high a detection limit of pIL-6, at which level the association may not be seen. Another recent meta-analysis failed to detect any association between pIL-6 and sarcopenia [33]. This latter review may have underestimated the influence of chronic diseases on pIL-6, since some of its studies reported extremely high pIL-6 in all participants, thus abolishing the standardized mean differences (SMD) between the sarcopenic and control groups [33]. Moreover, gender may also influence this association, since women with lower muscle strength have also shown lower (instead of higher) pIL-6 compared to men [8, 9, 34]. To date, no systematic review has analyzed this association separately in men and women. We therefore aimed to review the literature complemented by a meta-analysis to investigate the correlation between pIL-6 and muscle mass or strength in men and women.

## Methods

### Search strategy

The PRISMA principles [35] and MOOSE guidelines were followed. No review protocol was registered for this meta-analysis. The PubMed and Embase databases were systematically searched in August 2017 (see search strategy in Additional file 1). The following search terms were used: “interleukin-6” AND “muscle strength”; “interleukin-6” AND “muscle mass”. Articles were limited to human studies with participants over 65 years (mean or median value). Two hundred seventy-five records for “muscle strength” and 193 for “muscle mass” were identified altogether.

### Study selection

Selection was conducted separately by two investigators (AM and NF). Disagreements were resolved by a third reviewer (EP). Animal experiments, non-English-language reports and studies with all participants suffering from inflammatory diseases/states characterized by extremely high pIL-6 (e.g. renal failure, cancer, osteoarthritis, rheumatoid arthritis, chronic obstructive pulmonary diseases, sepsis, surgical interventions or cirrhosis) were excluded. Only groups without any intervention were included in two settings. Eligibility criteria for the first setting: presence of correlation coefficient regarding pIL-6 and any of the muscle parameters (muscle strength estimated by handgrip or knee extension or 4 m gait speed or chair stand or timed up and go or 6-min walk tests; muscle mass; fat-free mass; lean mass). Second setting: pIL-6 with at least one muscle parameter reported separately for male and female groups in the same study.

### Assessment of study quality

Strengthening the Reporting of Observational Studies in Epidemiology criteria (STROBE) [36] were used to assess the quality of the studies included. As few appropriate reports were available, we could not exclude studies based on lack of randomization, blinding or low participant number. Due to weighting methods, data with low participant numbers were assigned lower weights.

### Data extraction

Only data published in the original articles were extracted; no supplementary information was obtained. Data on patients with inflammatory diseases were excluded, but those of their controls were used. The following data were extracted: sample size, gender, age, mean (± standard deviation or standard error) or median (with quartiles) values of pIL-6 (converted to pg/mL) along with muscle parameters and correlation coefficients (if present).

### Data synthesis and analysis

A random effect model using the DerSimonian and Laird weighting was applied to account for study heterogeneity. To assess heterogeneity, the Q test and I^2^ indicator were calculated. A significant Q test (*p* < 0.1) or I^2^ higher than 50% indicated high heterogeneity. The estimated effect size (ES) was reported by the weighted mean with 95% confidence intervals (95% CI) and *p* < 0.05 was regarded as significant. To assess small study effect, we used Egger’s test to detect asymmetry in the funnel plot. A significant result (*p* < 0.1) indicates the existence of bias across studies.

With regard to correlations between pIL-6 and muscle parameters, the coefficients do not show normal distribution; therefore, Fisher’s z transformation was performed [37] with subgroup analyses of the male and female populations. Then, the results were back-transformed to correlation coefficients for interpretation purposes.

With regard to gender differences, we also analyzed male minus female differences of mean pIL-6 from studies that contained both male and female groups (effect size: paired difference). Gender differences in their age and muscle parameters were also tested. Due to differences in the methods and measurement scales for the assessment of muscle strength or mass, we had to use SMDs. For data on certain up and go and chair stand tests that were supplied in seconds (i.e. smaller values indicate better muscle performance), we reversed the sign of the difference. Medians with quartiles (not suitable for meta-analysis) were transformed to means with standard deviations [38].

Comprehensive Meta-Analysis Software V3.3 (Biostat Inc.) and Stata 11 SE (Stata Corp.) were employed.

## Results

### Study selection

**Fig. 1:**
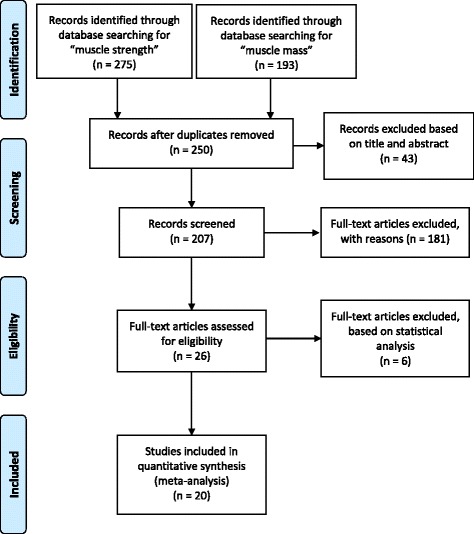
Flowchart of the study selection procedure

Two hundred fifty of the 468 items identified remained after duplicates were removed (Fig. 1). An initial screening of titles and abstracts removed non-English-language articles, animal studies, and those without full-text availability. A further 181 of the 207 full-text articles were excluded due to missing participant data, inappropriate age or comorbidities. The eligible 26 articles contained either (1) separate male and female data on pIL-6 along with corresponding muscle strength/mass values or (2) a correlation coefficient of association between pIL-6 and muscle parameters. Only the correlation coefficients for pIL-6 and handgrip strength (GS), the most widely used parameter, were analyzed, since numbers of studies reporting other muscle parameters were not statistically sufficient (knee extension test: 5; 4 m walk gait speed test: 2; 6-min walk test: 1; chair stand test: 3; muscle mass: 3). One study was excluded based on different mean ages for the male vs. female groups, and five studies were ruled out due to out-of-range correlation coefficients. The remaining 20 articles were included in the meta-analysis [14–18, 20, 23–25, 27–29, 31, 39–45] shown in the Table in Additional file 2.

### Study characteristics

Studies analyzed dated from 1995 to 2016. Sample size, gender, age and correlation coefficient and/or pIL-6 along with muscle strength/mass values for the same groups, provided separately for male and female populations, were extracted. Based on STROBE quality indicators (Additional file 3), we included all the data without exclusion.

Eleven [15–18, 20, 23–25, 27, 28, 31] of the 20 studies (involving a total of 3244 individuals ranging from 21 to 1020/study) investigated the correlation between pIL-6 and GS with 13 coefficients, since two analyzed correlations separately for male and female populations [24, 25]. Five studies [15–17, 20, 28] contained mixed data for male and female volunteers, while two studies each involved exclusively either men [18, 27] or women [23, 31].

Some authors reported a Pearson linear correlation [15, 18, 20, 23, 25, 28] sensitive only to linear associations, but others calculated the Spearman rank correlation [16, 24, 31], which measures any monotonous association. Both yield results between − 1 and 1. We used both types because this feature only affects the strength of the association, not its direction. Therefore, we only drew conclusions on the direction of the association. Correlations using a logarithmic [23, 25] or square root transformation [20] of pIL-6 were included, since these strictly monotonous transformations do not affect the direction of the association. Nine subgroups [15–18, 20, 23, 24, 27, 28] showed significant negative correlation, and only four failed to reveal any significant association [24, 25, 31].

Twelve of the 20 studies (involving 7209 individuals ranging from 10 to 1154/study, 3298 men and 3911 women) contained separate male and female data on pIL-6 with corresponding muscle parameters [14, 15, 24, 25, 29, 39–45]. One study [43] carried out the measurements in three different subgroups (with data for men and women, separately) providing independent data items. Male minus female differences in muscle parameters (GS, up and go test, chair stand test, knee extension test, 6-min walk test, fat-free mass, lean mass, thigh muscle area and muscle volume) and in age were also analyzed. With regard to muscle parameters, 26 pieces of data (male minus female SMD) were available, since individual studies provided up to three muscle parameters characterizing their male–female groups. Two studies [24, 44] that reported only the age range of the participants could not be used for an age comparison.

### Analysis of data and publication bias

**Fig. 2:**
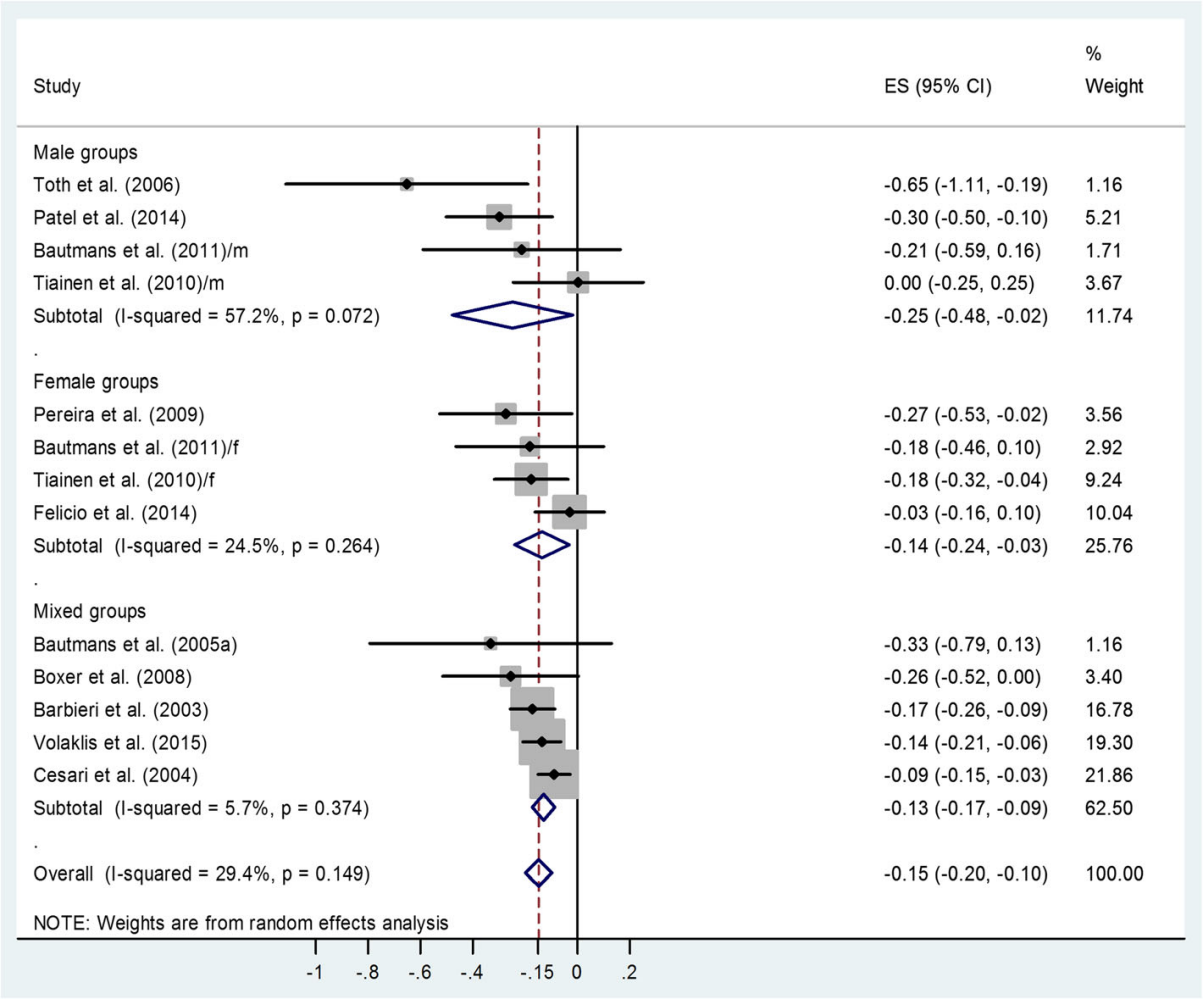
Forest plot representing Fisher’s z values concerning the relationship between pIL-6 level and grip strength in male, female and mixed subgroups. Squares show the Fisher’s z values with the grey area reflecting the weight assigned to the study. Horizontal bars indicate 95% confidence intervals (95% CI). The diamond shows the overall effect size (ES) with its corresponding 95% CI. In the case of two studies [24, 25], the coefficients for male and female groups were indicated by m and f, respectively

An analysis of the correlation coefficients using Fisher’s z transformation demonstrated a significant negative correlation between pIL-6 and GS in the 13 groups within the 11 studies (Fig. 2). To facilitate interpretation, weighted overall estimates (ES) were back-transformed to correlation coefficients: ES = − 0.15 with 95% confidence interval (CI) (− 0.20, − 0.10); *p* < 0.001. Little heterogeneity of data was found: *p* = 0.15, I^2^ = 29.4%. A small study effect was identified using Egger’s test: *p* = 0.044 for the correlation coefficient indicating some missing small studies with low correlation (funnel plot in Additional file 4). We also analyzed correlation coefficients separately for male and female subgroups (Fig. 2). The analysis demonstrated similarly negative correlations in women [23–25, 31] (ES = − 0.14 with 95% CI (− 0.24, − 0.03); *p* = 0.012) and men [18, 24, 25, 27] (ES = − 0.25 with 95% CI (− 0.48, − 0.02); *p* = 0.029). Subgroup analysis of male vs. female groups found no gender difference (Q = 0.123, *p* = 0.725).

Our results confirmed that pIL-6 negatively correlates with GS in both men and women. This confirmed negative correlation appears to suggest that men should have lower pIL-6 than women, because men have higher GS. However, it is not so. Some studies have reported that men with greater muscle strength also show higher pIL-6 than women of the corresponding age group [8, 9, 34]. These unexpected findings suggest that the range of pIL-6 is not the same in men and women.

**Fig. 3:**
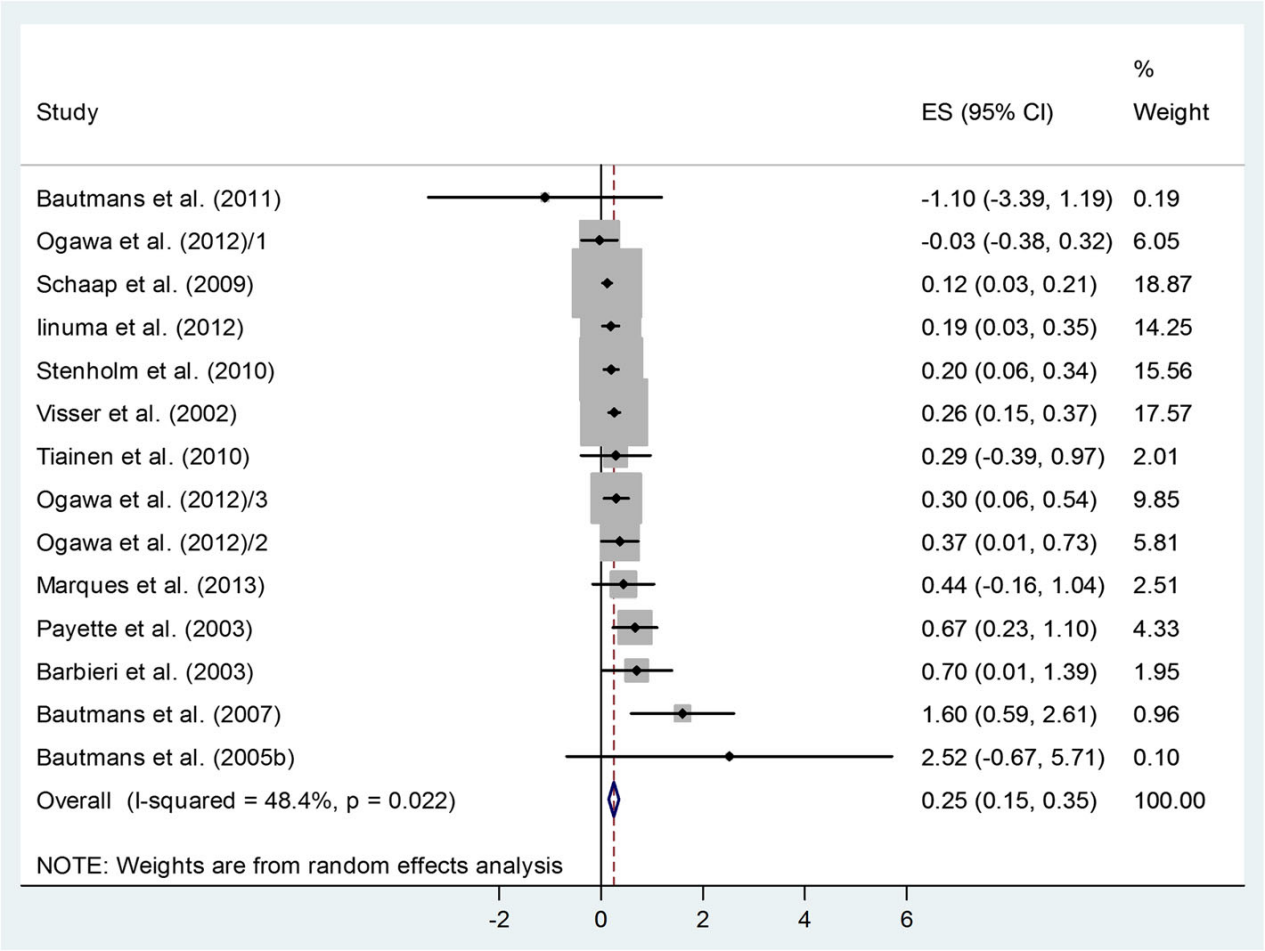
Forest plot representing the male minus female differences in pIL-6 from the same studies. Squares show the difference in mean values with the grey area reflecting the weight assigned to the study. Horizontal bars indicate 95% confidence intervals (95% CI). The diamond shows the overall effect size (ES) with the corresponding 95% CI. Subgroups in Ogawa et al. [43] are indicated with Arabic numerals

To test this hypothesis, we analyzed male minus female differences in mean pIL-6 in groups of similar age and different muscle conditions. In the 12 studies that contained male and female data, there was no significant difference in age (Additional file 5). At the same time, significantly better muscle condition was found in men (Additional file 6). A meta-analysis concerning male minus female differences in pIL-6 in groups from the same studies showed a significantly higher pIL-6 (Fig. 3) in men with better muscle condition: ES = 0.25 with 95% CI (0.15, 0.35); *p* < 0.001. The heterogeneity of the data was high: *p* = 0.022, I^2^ = 48.4%, indicating the contribution of other factors determining pIL-6. No small study effect was identified: *p* = 0.127.

## Discussion

We aimed to investigate the correlation between pIL-6 and muscle mass or strength in healthy elderly populations. Following the meta-analysis of the pooled data, correlation between pIL-6 and GS could be tested and it was analyzed separately in males and females. We also compared the male vs. female pIL-6 values from the same studies.

### Correlation between pIL-6 and grip strength

Our results showed a negative correlation between pIL-6 and GS in pooled and separate male and female elderly populations. The correlation coefficient was small but negative and similar in both genders. These results correspond with findings of the few available previous studies using other muscle parameters (muscle strength for knee flexion or extension [14, 18, 21, 26], 6-min walking distance [20], chair stand test [16], thigh [14] or leg muscle area [18]). Higher pIL-6 is associated not only with lower muscle mass/strength [14], but the higher level predicts a higher rate of muscle loss even in healthy elderly [46]. The most consistent relationship across different gender and race groups was observed for IL-6 and GS even after adjustment for age, health status, medications, physical activity, smoking, height and body fat [14]. Low physical performance is associated with low GS even after considering other risk factors for sarcopenia in the elderly, and low muscle strength has been reported to be a better indicator than low muscle mass [9]. GS is the most widely used indicator of sarcopenia in the elderly because this test is less influenced by age-related degeneration of the joints, yet it shows a strong correlation with upper and lower body strength and physical performance [2].

In contrast, some reports have failed to confirm this relationship. In populations with a uniformly low level of IL-6 [24, 31], a negative corrrelation may not be statistically detectable. Nevertheless, in our meta-analysis, the negative correlation has been proven, despite the low mean pIL-6 (below 2–2.5 pg/mL) in healthy individuals.

Proinflammatory cytokine IL-6 plays a central role in acute and chronic inflammatory diseases and geriatric syndromes (e.g. cardiovascular diseases, cancer, osteoporosis, chronic obstructive pulmonary disease, diabetes mellitus, Alzheimer’s disease and inflammatory bowel diseases) [4, 12, 19]. Other conditions, e.g. visceral obesity, smoking and stress, also trigger IL-6 release [47]. Nevertheless, several studies confirmed the negative correlation between pIL-6 and muscle strength (GS or quadriceps strength) independently of disease status even in such pathological states (e.g. chronic heart failure, obstructive lung disease) [18, 19, 22]. Potential mechanisms have been suggested by experimental studies, but their results are not conclusive. In some studies administration of IL-6 was reported to increase skeletal muscle protein breakdown and to decrease the rate of protein synthesis and muscle amino acid concentrations leading to muscle wasting in rats [48, 49], whereas other research groups demonstrated a lack of such effects of IL-6 in mice [50, 51]. In addition, a negative correlation has been found between IL-6 and endocrine regulators of muscle mass/strength, such as insulin-like growth factor-1 and dehydroepiandrosterone [52]. Based on these findings, a combination of high pIL-6, along with other inflammatory mediators and low levels of anabolic signals may contribute to the decline in muscle strength.

### Gender-related differences

The inverse correlation discussed above would suggest that women with lower muscle strength have a higher pIL-6 level than men with better muscle condition. However, certain studies reported lower IL-6 in elderly females than in males of similar age [8, 9, 34], while others failed to find any difference [24, 25]. Our meta-analysis of male minus female differences revealed significantly higher pIL-6 in men (despite better muscle condition) than in similarly elderly women (with weaker muscle condition). Different regulatory pathways of the female vs. male immune systems are suggested as an explanation. Moreover, gender difference may be observed in the age-related alterations of these regulatory pathways of the immune system [53]. Despite the similar negative correlations, males and females may be characterized by peculiar regression lines/curves. Therefore, blood samples collected from men should not be pooled with those of women. These findings may contribute to the explanation of the absence of a consensus cut-off point for the prediction of adverse physical and functional outcomes [15, 34]. Thus, in future clinical studies, a higher cut-off value for IL-6 must be defined for men than for women.

### Strengths and limitations

The main strength of our analysis is that we are the first to analyze male and female populations separately. Moreover, we selected studies that recorded resting pIL-6, as pIL-6 level is transiently increased following exercise.

Limitations mainly stem from features of available studies. Studies that pooled samples of men and women also had to be included, whereas only a few reports were appropriate for a gender-based analysis of correlation. The small study effect with regard to correlation coefficients represents another limitation. In most studies, only mean age was supplied for a wide age range of participants, limiting the strength of an analysis of age. In addition, high heterogeneity with regard to gender difference in pIL-6 indicates the presence of other determining factors, e.g. diet, obesity, smoking, stress, undiagnosed inflammatory processes, different sample techniques and assay methods. Although our analysis focused on healthy elderly individuals, sporadic inflammatory conditions could not be completely excluded.

## Conclusions

The plasma level for IL-6 correlates negatively and reportedly in a nonlinear fashion [34] with muscle strength; thus it is a potential biomarker of sarcopenia. The practical implication of our study is that a different interpretation of IL-6 as a biomarker of aging sarcopenia is needed in men and women in future studies. Accordingly, higher IL-6 cut-off values must be determined in men than in women with regard to aging sarcopenia.

## Additional files

Additional file 1: Literature search strategy. (DOCX 14 kb)

Additional file 2: Description of the studies included in the meta-analysis (*n* = 20). (DOCX 22 kb)

Additional file 3: Quality assessment of the papers (observational studies) included based on guidelines from the STROBE statement. (DOCX 16 kb)

Additional file 4: Funnel plot of standard error using Fisher’s z. The asymmetrical funnel plot represents the small study effect with regard to Fisher’s z values derived from correlation coefficients. (TIF 40 kb)

Additional file 5: Forest plot representing the male minus female differences in mean age of corresponding groups from the same studies. Squares show the difference in mean values with the grey area reflecting the weight assigned to the study. Horizontal bars indicate 95% confidence intervals (95% CI). The diamond shows the overall effect size (ES) with the corresponding 95% CI. Subgroups in Ogawa et al. [43] are indicated with Arabic numerals. A lack of age difference was found: ES = 0.14 with 95% CI (− 0.11, 0.39) *p* = 0.265. The heterogeneity of the data was low: *p* = 0.195, I^2^ = 25.3%. No small study effect was identified using Egger’s test: *p* = 0.487. (TIF 156 kb)

Additional file 6: Forest plot representing the male minus female standardized differences in mean values of various muscle parameters (muscle mass/strength/function) in corresponding groups from the same studies. Squares show the difference in mean values with the grey area reflecting the weight assigned to the study. Horizontal bars indicate 95% confidence intervals (95% CI). The diamond shows the overall effect size (ES) with the corresponding 95% CI. Subgroups in Ogawa et al. [43] are indicated with Arabic numerals. A: Grip strength; B: Chair stand test; C: 6-min walk; D: Lean mass; E: Up and go test; F: Muscle volume; G: Fat-free mass; H: Knee extension test; I: Thigh muscle area. Significantly higher muscle mass/strength/function was demonstrated in male than in female volunteers (i.e. positive standardized difference in mean values): ES = 1.82 with 95% CI (1.62, 2.02) *p* < 0.001. The heterogeneity of the data was high: *p* < 0.001, I^2^ = 95.4%. No small study effect was identified using Egger’s test: *p* = 0.163. (TIF 295 kb)

## Abbreviations

CI: Confidence interval
ES: Effect size
GS: Grip strength
pIL-6: plasma interleukin-6
PRISMA: Preferred reporting items for systematic review and meta-analysis
SMD: Standardized mean difference
STROBE: Strengthening the Reporting of Observational Studies in Epidemiology
