# Supplementary material for: Gender difference in the effects of interleukin-6 on grip strength – a systematic review and meta-analysis
Source: BMC Geriatr. 2018 May 8;18:107. doi: 10.1186/s12877-018-0798-z (PMC5941705; doi:10.1186/s12877-018-0798-z)
Supplement: Supplementary file 1 — Literature search strategy. (DOCX 14 kb) [file 12877_2018_798_MOESM1_ESM.docx]

**Additional File 1.** Literature search strategy

Relevant studies, published before August 01, 2017 (date last searched), were identified through electronic searches limited to the English language using MEDLINE and EMBASE databases. Electronic searches were supplemented by scanning reference lists of articles identified for all relevant studies (including review articles).

Limitations: Humans; English; Age: 65+ years

**PUBMED**

http://www.ncbi.nlm.nih.gov/pubmed

1)

("**muscle strength**"[MeSH Terms] OR ("muscle"[All Fields] AND "strength"[All Fields]) OR "muscle strength"[All Fields]) AND ("interleukin-6"[MeSH Terms] OR "interleukin-6"[All Fields] OR "interleukin 6"[All Fields]) AND ("humans"[MeSH Terms] AND English[lang] AND "aged"[MeSH Terms])

2)

"**muscle mass**"[All Fields] AND ("interleukin-6"[MeSH Terms] OR "interleukin-6"[All Fields] OR "interleukin 6"[All Fields]) AND ("humans"[MeSH Terms] AND English[lang] AND "aged"[MeSH Terms])

**EMBASE**

https://www.embase.com

In Embase we excluded editorials, erratums, letters and notes.

1)

'**muscle strength**' AND 'interleukin-6' AND [aged]/lim AND [humans]/lim AND [english]/lim AND ([article]/lim OR [article in press]/lim OR [conference abstract]/lim OR [conference paper]/lim OR [conference review]/lim OR [review]/lim OR [short survey]/lim)

2)

'**muscle mass**' AND 'interleukin 6' AND [humans]/lim AND [english]/lim AND [aged]/lim AND ([article]/lim OR [article in press]/lim OR [conference abstract]/lim OR [conference paper]/lim OR [conference review]/lim OR [review]/lim OR [short survey]/lim)
