# Supplementary material for: Gender difference in the effects of interleukin-6 on grip strength – a systematic review and meta-analysis
Source: BMC Geriatr. 2018 May 8;18:107. doi: 10.1186/s12877-018-0798-z (PMC5941705; doi:10.1186/s12877-018-0798-z)
Supplement: Supplementary file 2 — Description of the studies included in the meta-analysis (n = 20). (DOCX 22 kb) [file 12877_2018_798_MOESM2_ESM.docx]

**Additional file 2 (Table):** Description of the studies included in the meta-analysis

| **Study [number in the List of References]** | **Reference code** | **Sample size** | **Gender** | **Age** | **Measured muscle parameter (unit)** | **Value of measured muscle parameter** | **IL-6 (pg/mL)** | **correlation coefficient (IL-6 vs. grip strength)** |
| --- | --- | --- | --- | --- | --- | --- | --- | --- |
| Barbieri et al. (2003)[15] | 15/A | 222 | male | 65±15 (mean±SD) | grip strength (kg) | 41.9±11.9 (mean±SD) | 2.45±5.71 (mean±SD) | - |
|  | 15/A | 304 | female | 66±16 (mean±SD) | grip strength (kg) | 25.2±7.6  (mean±SD) | 1.75±1.88 (mean±SD) | - |
|  |  | 526 | mixed | - | - | - | - | -0.17 |
| Bautmans et al. (2005a)[17] |  | 63 21m/42f | mixed | 84.2±5.7 (mean±SD) | - | N/A | N/A | -0.32 |
| Bautmans et al. (2005b)[39] | 39/A | 10 | male | 69.3±5.6 (mean±SD) | grip strength (kPa) | 83.9±19 (mean±SD) | 5.21±6.91 (mean±SD) | - |
|  | 39/B |  |  |  | chair stand test (rep/30s) | 14.5±4.6 (mean±SD) |  | - |
|  | 39/C |  |  |  | 6-minute walk (m) | 605.8± 74 (mean±SD) |  | - |
|  | 39/A | 21 | female | 68±6 (mean±SD) | grip strength (kPa) | 60.7±14.9 (mean±SD) | 2.69±2.14 (mean±SD) | - |
|  | 39/B |  |  |  | chair stand test (rep/30s) | 15.6±4 (mean±SD) |  | - |
|  | 39/C |  |  |  | 6-minute walk (m) | 550.1± 85.3 (mean±SD) |  | - |
| Bautmans et al. (2007)[40] | 40/A | 25 | male | 75.6 ± 5.2 (mean±SD) | grip strength (kPa) | 73.0 ± 17.2 (mean±SD) | 4.9 ± 1.8 (mean±SD) | - |
|  | 40/A | 15 | female | 75.3 ± 6.0 (mean±SD) | grip strength (kPa) | 54.4 ± 13.0 (mean±SD) | 3.3 ± 1.1 (mean±SD) | - |
| Bautmans et al. (2011)[25] | 25/A | 49 | male | 74.7±4.7 (mean±SD) | grip strength (kPa) | 74.3±17.6 (mean±SD) | 5.1±5.8 (mean±SD) | - |
|  | 25/A | 51 | female | 74.2±4.5 (mean±SD) | grip strength (kPa) | 49.9±12.3 (mean±SD) | 6.2±5.9 (mean±SD) | - |
|  | 25/m | 79 | male | - | - | - | - | -0.21 |
|  | 25/f | 112 | female | - | - | - | - | -0.18 |
| Boxer et al. (2008)[20] |  | 60 43m/17f | mixed | 77±10 (mean±SD) | - | N/A | N/A | -0.25 |
| Cesari et al. (2004)[16] |  | 1020 447m/573f | mixed | 75.4±0.2 (mean±SE) | - | N/A | N/A | -0.089 |
| Felicio et al. (2014)[31] |  | 221 | female | 71.07±4.93 (mean±SD) | - | N/A | N/A | -0.03 |
| Iinuma et al. (2012)[41] | 41/A | 219 | male | 87.3 (86.1–88.8) (median, quartiles) | grip strength (kg) | 24.7 (21.3-27.3) (median, quartiles) | 1.8 (1.36-2.60) (median, quartiles) | - |
|  | 41/A | 270 | female | 87.3 (86.4–88.9) (median, quartiles) | grip strength (kg) | 16.3 (14-19) (median, quartiles) | 1.57 (1.23-2.40) (median, quartiles) | - |
| Marques et al. (2013)[42] | 42/B | 23 | male | 68.2±5.2 (mean±SD) | chair stand test (rep/30s) | 18.65±4.27 (mean±SD) | 1.62±1.25 (mean±SD) | - |
|  | 42/D |  |  |  | lean mass (kg) | 54.9±5.9 (mean±SD) |  | - |
|  | 42/E |  |  |  | up and go test (s) | 4.55±0.74 (mean±SD) |  | - |
|  | 42/B | 24 | female | 68.2±5.7 (mean±SD) | chair stand test (rep/30s) | 18.54±4.48 (mean±SD) | 1.18±0.81 (mean±SD) | - |
|  | 42/D |  |  |  | lean mass (kg) | 38.4±4.8 (mean±SD) |  | - |
|  | 42/E |  |  |  | up and go test (s) | 5.33±1.04 (mean±SD) |  | - |
| Ogawa et al. (2012)[43] | 43/1A | 109 | male | 72 (68–77) (median, quartiles) | grip strength (kg) | 34.5 (30.0-38.5) (median, quartiles) | 2.1 (1.5-2.7) (median, quartiles) | - |
|  | 43/1F |  |  |  | muscle volume (kg) | 24.7 (22.8–27.1) (median, quartiles) |  | - |
|  | 43/1A | 120 | female | 71 (67–78) (median, quartiles) | grip strength (kg) | 20 (16.8-22.5) (median, quartiles) | 1.7 (1.2-2.5) (median, quartiles) | - |
|  | 43/1F |  |  |  | muscle volume (kg) | 17.8 (15.6–19.9) (median, quartiles) |  | - |
|  | 43/2A | 85 | male | 72 (68–77) (median, quartiles) | grip strength (kg) | 33 (28.5-37) (median, quartiles) | 2.1 (1.3-3.4) (median, quartiles) | - |
|  | 43/2F |  |  |  | muscle volume (kg) | 24.3 (22.2–26.4) (median, quartiles) |  | - |
|  | 43/2A | 122 | female | 71 (68–76) (median, quartiles) | grip strength (kg) | 21 (16.8-24) (median, quartiles) | 1.9 (1.2-2.6) (median, quartiles) | - |
|  | 43/2F |  |  |  | muscle volume (kg) | 18 (16.2–19.5) (median, quartiles) |  | - |
|  | 43/3A | 76 | male | 73 (69–79) (median, quartiles) | grip strength (kg) | 32.5 (26.5-37) (median, quartiles) | 2 (1.4-2.8) (median, quartiles) | - |
|  | 43/3F |  |  |  | muscle volume (kg) | 24 (21.5–26) (median, quartiles) |  | - |
|  | 43/3A | 140 | female | 74 (69–78) (median, quartiles) | grip strength (kg) | 19.5 (16.5-22.5) (median, quartiles) | 1.9 (1.3-3.1) (median, quartiles) | - |
|  | 43/3F |  |  |  | muscle volume (kg) | 17.8 (15.9–18.9) (median, quartiles) |  | - |
| Patel et al. (2014)[27] |  | 96 | male | 72.3±2.41 (mean±SD) | - | N/A | N/A | -0.29 |
| Payette et al. (2003)[44] | 44/G | 232 | male | 78 (72-92) (mean, range) | fat free mass (kg) | 55.1±5.8 (mean±SD) | 4.3 (3.1–7.2) (median, quartiles) | - |
|  | 44/G | 323 | female |  | fat free mass (kg) | 38.1±4.1 (mean±SD) | 3.5 (3.1–6.0) (median, quartiles) | - |
| Pereira et al. (2009)[23] |  | 63 | female | 71.2± 7.4 (mean±SD) | - | N/A | N/A | -0.2673 |
| Schaap et al. (2009)[29] | 29/A | 1023 | male | 73.6 ± 2.8 (mean±SD) | grip strength (kg) | 39±8.2 (mean±SD) | 1.8 (1.27-2.67) (median, quartiles) | - |
|  | 29/H |  |  |  | knee extensor strength (Nm) | 135.3 ± 34.9 (mean±SD) |  | - |
|  | 29/I |  |  |  | thigh muscle area (cm^2^) | 264.7±42.5 (mean±SD) |  | - |
|  | 29/A | 1154 | female | 73.3 ± 2.8 (mean±SD) | grip strength (kg) | 23.75±5.7 (mean±SD) | 1.66 (1.14-2.57) (median, quartiles) | - |
|  | 29/H |  |  |  | knee extensor strength (Nm) | 82.7 ± 22.2 (mean±SD) |  | - |
|  | 29/I |  |  |  | thigh muscle area (cm^2^) | 184.8±34.2 (mean±SD) |  | - |
| Stenholm et al. (2010)[45] | 45/A | 310 | male | 73.1±6.4 (mean±SD) | grip strength (kg) | 38.8±10.2 (mean±SD) | 1.5 (0.9-2.3) (median, quartiles) | - |
|  | 45/A | 406 | female | 74.2±6.6 (mean±SD) | grip strength (kg) | 22.2±7.6 (mean±SD) | 1.3 (0.8-2.0) (median, quartiles) | - |
| Tiainen et al. (2010)[24] | 24/A, 24/m | 65 | male | 90< | grip strength (kg) | 46.00 (36.00–52.50)  (median, quartiles) | 2.56 (1.71–5.42) (median, quartiles) | 0.003 |
|  | 24/B |  |  |  | chair stand test (s) | 18.00 (14.00–20.00)  (median, quartiles) |  | N/A |
|  | 24/A, 24/f | 197 | female | 90< | grip strength (kg) | 28.00 (18.50–40.00)  (median, quartiles) | 2.59 (1.57–4.65) (median, quartiles) | -0.175 |
|  | 24/B |  |  |  | chair stand test (s) | 20.00 (15.00–25.00)  (median, quartiles) |  | N/A |
| Toth et al. (2006)[18] |  | 21 | male | 70±3 (mean±SE) | - | - | - | -0.573 |
| Visser et al. (2002)[14] | 14/A | 850 | male | 73.9±2.9 (mean±SD) | grip strength (kg) | 37.5±7.5 (mean±SD) | 1.84 (1.31-2.83) (median, quartiles) | - |
|  | 14/H |  |  |  | knee extensor strength (Nm) | 130.9±33.4 (mean±SD) |  | - |
|  | 14/I |  |  |  | thigh muscle area (cm^2^) | 254.6±38.5) (mean±SD) |  | - |
|  | 14/A | 764 | female | 73.5±2.8 (mean±SD) | grip strength (kg) | 22.4±5.05 (mean±SD) | 1.63 (1.10-2.47) (median, quartiles) | - |
|  | 14/H |  |  |  | knee extensor strength (Nm) | 78.5±19.5 (mean±SD) |  | - |
|  | 14/I |  |  |  | thigh muscle area (cm^2^) | 170.6±27.6 (mean±SD) |  | - |
| Volaklis et al. (2015)[28] |  | 721 353m/398f | mixed | 75.4±6.6 (mean±SD) | - | N/A | N/A | -0.135 |

Interleukin-6, IL-6; N/A, Not applicable; SD, Standard deviation; SE, Standard error; A, Grip strength; B, Chair stand test; C, 6-minute walk; D, Lean mass; E, Up and go test; F, Muscle volume; G, Fat free mass; H, Knee extension test; I, Thigh muscle area; f, female; m, male;

One study [43] carried out the measurements in three different subgroups indicated by 43/1, 2, 3.
