# Supplementary material for: Gender difference in the effects of interleukin-6 on grip strength – a systematic review and meta-analysis
Source: BMC Geriatr. 2018 May 8;18:107. doi: 10.1186/s12877-018-0798-z (PMC5941705; doi:10.1186/s12877-018-0798-z)
Supplement: Supplementary file 3 — Quality assessment of the papers (observational studies) included based on guidelines from the STROBE statement. (DOCX 16 kb) [file 12877_2018_798_MOESM3_ESM.docx]

**Additional file 3.** Quality assessment of observational studies based on guidelines from the STROBE statement.

| Study | Clear Description of Participant Eligibility and Sources / Methods of Participant Selection | Clearly Defined Settings | | Clearly Defined Outcomes | | Clear Description of Handling of Muscle Parameters and Plasma Interleukin-6 in the Analyses | Control for Potential Confounders by Exclusion or Statistical Adjustment |
| --- | --- | --- | --- | --- | --- | --- | --- |
|  |  | Ascertainment: Correlation Analysis between Plasma Interleukin -6 and Grip Strength | Ascertainment: Clearly Defined Aged Female and Male Groups | Ascertainment: Correlation Coefficient between Plasma Interleukin -6 and Grip Strength | Ascertainment: Muscle Parameters and Plasma Interleukin-6 |  |  |
| Barbieri et al. (2003) | X | X | X | X | X | X | X |
| Bautmans et al. (2005a) | X | X |  | X |  | X | X |
| Bautmans et al. (2005b) | X |  | X |  | X | X | X |
| Bautmans et al. (2007) | X |  | X |  | X | X | X |
| Bautmans et al. (2011) | X | X | X | X | X | X | X |
| Boxer et al. (2008) | X | X |  | X |  | X | X |
| Cesari et al. (2004) | X | X |  | X |  | X | X |
| Felicio et al. (2014) | X | X |  | X |  | X | X |
| Iinuma et al. (2012) | X |  | X |  | X | X | X |
| Marques et al. (2013) | X |  | X |  | X | X | X |
| Ogawa et al. (2012) | X |  | X |  | X | X | X |
| Patel et al. (2014) | X | X |  | X |  | X | X |
| Payette et al. (2003) | X |  | X |  | X | X | X |
| Pereira et al. (2009) | X | X |  | X |  | X | X |
| Schaap et al. (2009) | X |  | X |  | X | X | X |
| Stenholm et al. (2010) | X |  | X |  | X | X | X |
| Tiainen et al. (2010) | X | X | X | X | X | X | X |
| Toth et al. (2006) | X | X |  | X |  | X | X |
| Visser et al. (2002) | X |  | X |  | X | X | X |
| Volaklis et al. (2015) | X | X |  | X |  | X | X |

From: E. von Elm, D.G. Altman, M. Egger, S.J. Pocock, P.C. Gotzsche, J.P.Vandenbroucke, The strengthening the reporting of observational studies in epidemiology (STROBE) statement: guildelines for reporting observational studies, J. Clin. Epidemiol. 61 (2008) 344-349. doi: 10.1016/j.jclinepi.2007.11.0
