# Supplementary figures and images for: Gender difference in the effects of interleukin-6 on grip strength – a systematic review and meta-analysis
Source: BMC Geriatr. 2018 May 8;18:107. doi: 10.1186/s12877-018-0798-z (PMC5941705; doi:10.1186/s12877-018-0798-z)

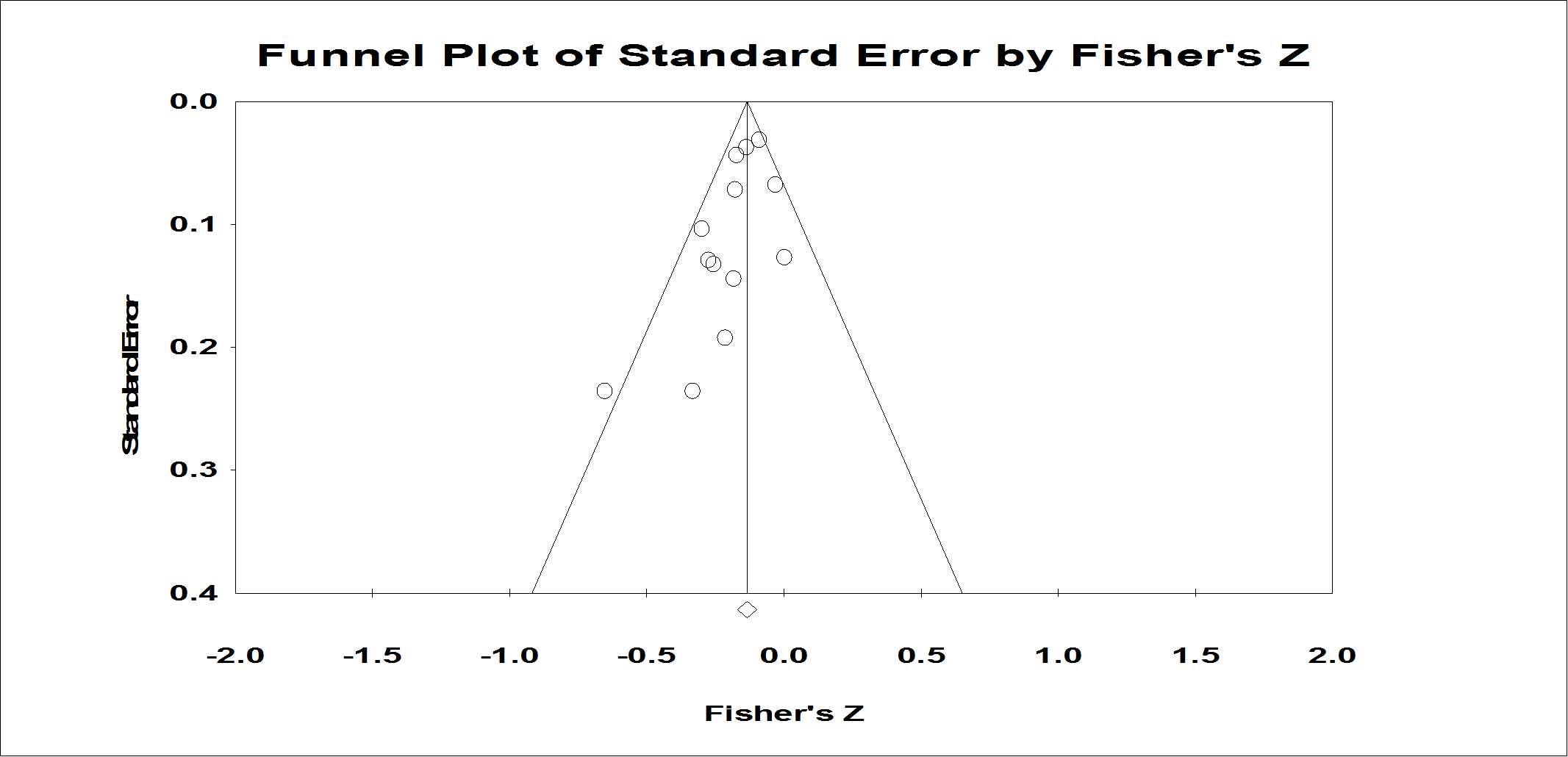

Supplement: Supplementary file 4 — Funnel plot of standard error using Fisher’s z. The asymmetrical funnel plot represents the small study effect with regard to Fisher’s z values derived from correlation coefficients. (TIF 40 kb) [file 12877_2018_798_MOESM4_ESM.tif]

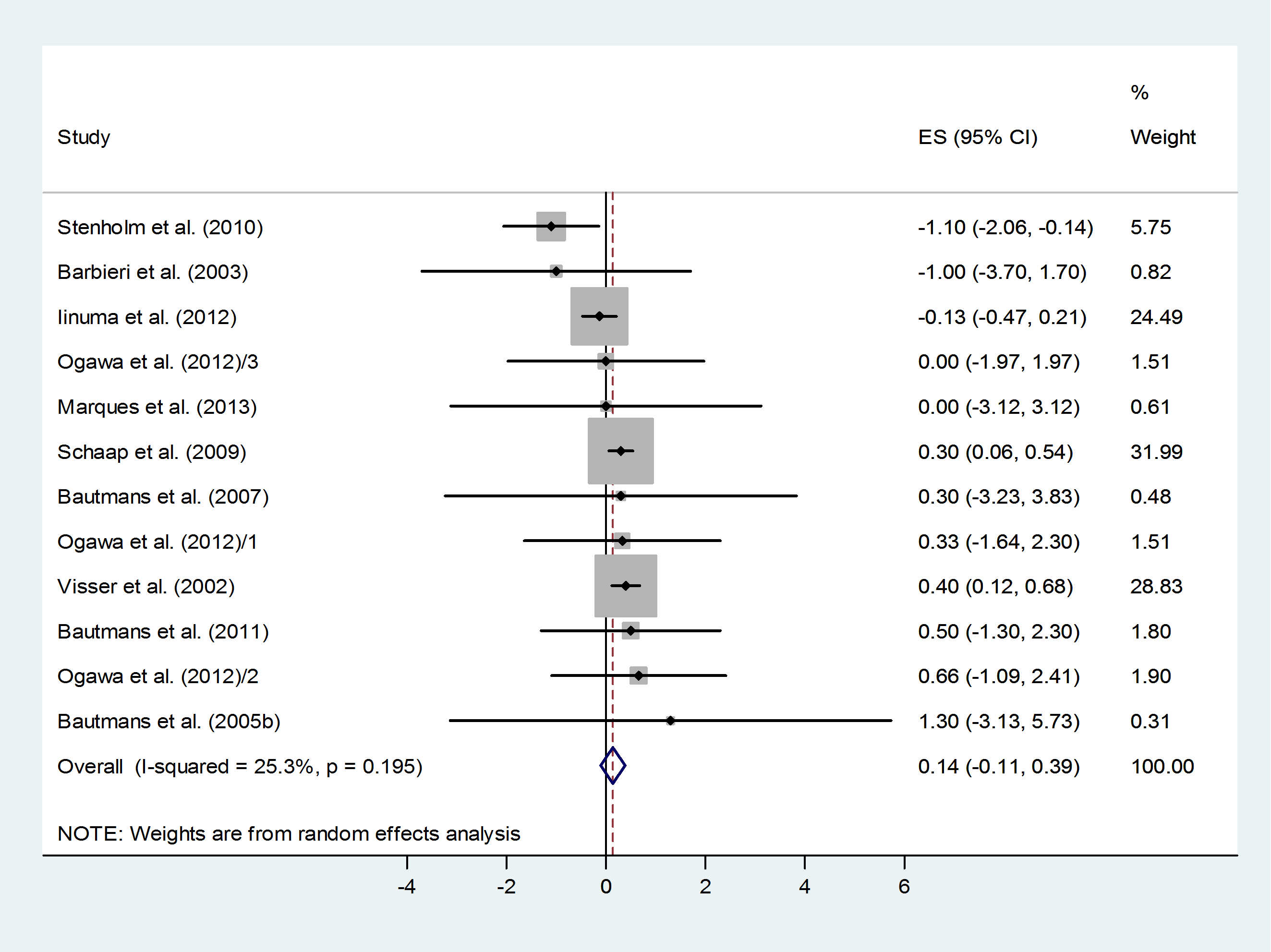

Supplement: Supplementary file 5 — Forest plot representing the male minus female differences in mean age of corresponding groups from the same studies. Squares show the difference in mean values with the grey area reflecting the weight assigned to the study. Horizontal bars indicate 95% confidence intervals (95% CI). The diamond shows the overall effect size (ES) with the corresponding 95% CI. Subgroups in Ogawa et al. [43] are indicated with Arabic numerals. A lack of age difference was found: ES = 0.14 with 95% CI (− 0.11, 0.39) p = 0.265. The heterogeneity of the data was low: p = 0.195, I2 = 25.3%. No small study effect was identified using Egger’s test: p = 0.487. (TIF 156 kb) [file 12877_2018_798_MOESM5_ESM.tif]

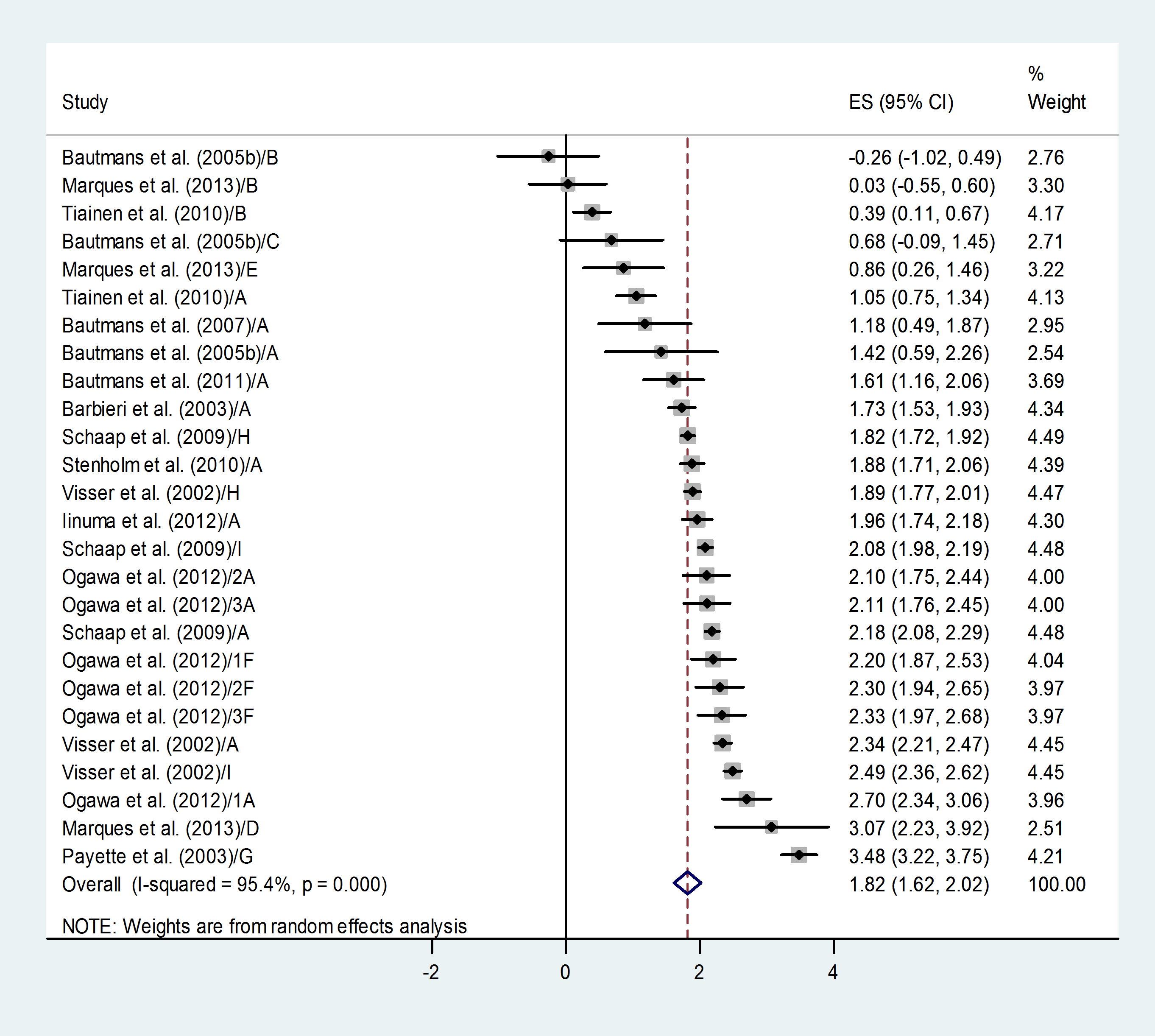

Supplement: Supplementary file 6 — Forest plot representing the male minus female standardized differences in mean values of various muscle parameters (muscle mass/strength/function) in corresponding groups from the same studies. Squares show the difference in mean values with the grey area reflecting the weight assigned to the study. Horizontal bars indicate 95% confidence intervals (95% CI). The diamond shows the overall effect size (ES) with the corresponding 95% CI. Subgroups in Ogawa et al. [43] are indicated with Arabic numerals. A: Grip strength; B: Chair stand test; C: 6-min walk; D: Lean mass; E: Up and go test; F: Muscle volume; G: Fat-free mass; H: Knee extension test; I: Thigh muscle area. Significantly higher muscle mass/strength/function was demonstrated in male than in female volunteers (i.e. positive standardized difference in mean values): ES = 1.82 with 95% CI (1.62, 2.02) p < 0.001. The heterogeneity of the data was high: p < 0.001, I2 = 95.4%. No small study effect was identified using Egger’s test: p = 0.163. (TIF 295 kb) [file 12877_2018_798_MOESM6_ESM.tif]
